# Circulating tumor DNA kinetics: A future tool for radiation therapy personalization in lung cancer?

**DOI:** 10.1016/j.jlb.2024.100160

**Published:** 2024-06-21

**Authors:** Gerard M. Walls, Bruna Pellini, Aadel A. Chaudhuri

**Affiliations:** a Department of Radiation Oncology, Washington University in St Louis, 4511 Forest Park Avenue, Saint Louis, MO, 63108, United States; b Patrick Johnston Centre for Cancer Research, Queen's University Belfast, Jubilee Road, Belfast, BT9 7AB, UK, Northern Ireland, UK; c Department of Thoracic Oncology, Moffitt Cancer Center and Research Institute, Tampa, FL, 33612, United States; d Department of Oncologic Sciences, Morsani College of Medicine, University of South Florida, Tampa, FL, 33612, United States; e Department of Radiation Oncology, Mayo Clinic, Rochester, MN, United States; f Mayo Clinic Comprehensive Cancer Center, Rochester, MN, United States

**Keywords:** Lung cancer, Radiation therapy, ctDNA, Biomarker, Personalization

## Abstract

Locally advanced non-small cell lung cancer (NSCLC) is a highly diverse disease in terms of its histology, staging and molecular biology, yet chemoradiation treatment paradigms are currently not individualized for these factors. Circulating tumor DNA (ctDNA) in the plasma has clinical utility for several systemic therapies in advanced NSCLC, before, during and after treatment. However, in radiation oncology, the evidence for ctDNA in locally advanced NSCLC is largely limited to minimal residual disease (MRD) detection after treatment. In one study, all post-treatment MRD-positive patients developed disease relapse, compared with just 7% of ctDNA MRD-negative patients. As chemoradiation causes a bolus of tumor cell death with each treatment fraction, it has been posited that ctDNA levels fluctuate as well on a per-fraction basis. Indeed, four preliminary studies reveal a subgroup of patients with a transient elevation of ctDNA levels after the first radiotherapy fraction, followed by an overall ctDNA drop. To follow-up on these data suggesting an early ctDNA spike during chemoradiation, there is a need for larger and more systematic inter-fraction ctDNA studies, which if successful, could form the basis for more personalized and efficacious radiotherapy treatment paradigms.

## 1 Background

The majority of patients with unresectable locally advanced non-small cell lung cancer (NSCLC) develop relapse despite curative-intent chemoradiation and adjuvant durvalumab [1]. Aggressive management options for relapse have emerged, such as re-irradiation [2,3] and immunotherapy [4], but surveillance is limited to clinical assessment and imaging, which may not be sensitive enough. Tumor-derived fragments of cell-free DNA, known as circulating tumor DNA (ctDNA), are widely used by medical oncologists for noninvasive molecular profiling, and hold promise for on-treatment immunotherapy response monitoring [5]. In contrast, ctDNA has not been integrated into routine radiation oncology care to date [6], largely related to the smaller quantities of ctDNA associated with localised tumors suitable for definitive-intent radiation therapy (RT), and the current lack of literature to guide RT personalization based on tumor genomic alterations.

## 2 Current applications in NSCLC radiation therapy

The indication with the most data in RT for NSCLC is minimal residual disease (MRD) detection after curative-intent treatment. In 2017, the first study of NSCLC patients undergoing RT demonstrated that the presence of ctDNA within 4 months of treatment completion was associated with shorter progression-free and lung cancer-specific survival [7]. All MRD-positive patients developed disease relapse, compared with just 7% of MRD-negative patients. Longitudinal surveillance with ctDNA revealed 100% sensitivity and specificity for future recurrence, and often preceded radiological relapse by ​> ​5 months, with subsequent studies largely confirming these findings. In 2023, a large real-world study investigated the prognostic value of pre-treatment ctDNA assessment in 309 patients undergoing RT for oligometastatic NSCLC [8]. In this retrospective multi-institutional study, detectable pre-RT ctDNA was associated with worse progression-free and overall survival. This exciting data is the first hint that ctDNA could be used for risk-stratification to aid patient selection for aggressive RT in oligometastatic NSCLC.

## 3 ctDNA kinetics during radiation therapy

As RT is known to cause cell death in the acute setting, it has been hypothesised that ctDNA levels may fluctuate when treatment is initiated. Two proof-of-concept studies demonstrated this principle in NSCLC in 2020 [9,10], and have been corroborated subsequently [11,12] (Table 1). These studies suggest that the levels of detectable ctDNA increase after the initiation of RT in a third of patients, with peaks occurring in hours-to-days following the first treatment fraction. In most patients, the ctDNA peak was quickly followed by a decline, resembling a ‘spike’. The impact of increasing fraction size or adding concurrent chemotherapy is not ascertainable in these small sample sizes, but both could plausibly lead to more pronounced spikes via enhanced tumor cell kill. Real-time on-treatment ctDNA levels could also be affected by physiological factors including circulating nuclease activity and renal excretion. As such, person-to-person variability in the time taken for ctDNA clearance may also warrant further investigation. That ctDNA negativity at a single ‘on-treatment’ timepoint (4 weeks) was prognostically slightly superior (progression-free survival) to a single post-RT sample (≤3 days after RT completion) in a large Chinese study is provocative [13]. Still, drawing concrete conclusions from all the available data is challenging due to the heterogeneity in treatment paradigms, blood collection time-points, and ctDNA assays. An ongoing large prospective cohort study in the UK with serial sampling during treatment and ultra-deep sequencing, VIGILANCE (NCT06086574), is likely to provide high quality data in the near future.

**Table 1:** A summary of published inter-fraction ctDNA kinetics studies.

Study	n	RT Dose Fractionation	Concurrent Chemotherapy	Time Points	Assay Approach	Reported assay lower limit of detection (%)^a^	Summary of Findings
Nygård et al. 2020^9^	6	Daily 2Gy x 33	Yes (C1 given 3 weeks pre-#1)	• At diagnosis	Shallow whole genome sequencing	1	3/6 patients had higher ctDNA levels at #11 compared to #1
				• Planning scan			
				• 30 ​min post-#1			
				• 30 ​min post-#11			
Walls et al. 2020^10^	5	Daily 2.75Gy x 20	None	• Pre-#1	77-gene NGS panel	0.5–1.0	2/5 patients had higher ctDNA levels at 72 hours compared to baseline, followed by a drop
				• Pre-#3			
				• Pre-#6			
Breadner et al. 2022^11^	10	Daily 2Gy x 27–33	Yes (C1 immediately pre-#1)	• Within 2 weeks	36-gene NGS panel	0.06	Patient-level data not provided but ctDNA spike was observed across the group (median time of 4 hours)
				• Pre-#1			
				• 3 ​h post-#1			
				• 6 ​h post-#1			
				• Post-#2			
				• Post-#3			
MacManus et al. 2023^12^	12	Daily 2Gy x 30 or Daily 2.75Gy x 20	Yes (C1 timing NR)	• Baseline	ddPCR	NR	3/12 patients had higher ctDNA levels at 24 hours compared to baseline, followed by a drop
				• 24-h post-RT			
				• 4–6 weeks post-final #			
				• Later variable time-point			

(n ​= ​number of patients included receiving fractionated radiotherapy; Gy ​= ​Gray; C1 ​= ​cycle one; # ​= ​radiotherapy fraction number; NGS ​= ​next-generation sequencing; ddPCR ​= ​droplet digital polymerase chain reaction; NR ​= ​not reported).

a = ​based on VAF calculation or tumor fraction inference.

## 4 Outstanding barriers to ctDNA monitoring during radiation

Before the potential of ctDNA kinetics during RT can be elucidated, several aspects of this approach require significant refinement. The clinical priority is to establish optimal time-points for blood collection in NSCLC, to avoid under-estimation of ctDNA detection and to assist inter-trial comparisons. Reporting should be standardised, in terms of the precise timing of sample collection (i.e., hours or days) in relation to the treatment delivery±subsequent fractions. Accounting for ‘weekend effects’ will also be essential, as well as the administration timing of induction and concurrent cytotoxic agents or immunotherapy. For patients receiving hypofractionated treatment, alternate day radiation delivery can be expected to influence ctDNA kinetics, too.

The technical priority is assay sensitivity, particularly for patients with low-volume disease, since how early-stage NSCLC is associated with much lower ctDNA levels and detection rates than metastatic disease is well established [[14], [15], [16]]. Linked with sensitivity, there is ongoing debate regarding the merits of tumor-informed versus tumor-uninformed assays. Tumor-informed assays may be more expensive and results may take longer, as they require up-front next-generation sequencing of tumor samples with subsequent development of a personalized assay with individualized probes. However, these assays typically account for clonal haematopoietic mutations of indeterminate potential (CHIP) [17] by sequencing both tumor and plasma-depleted whole blood at the initial timepoint, therefore yielding potentially improved assay sensitivity and specificity. However, these assays may miss *de novo* mutations that arise during the course of treatment.

In relation to conducting gold-standard clinical studies, the major hurdle to overcome is identifying a CLIA-approved assay with optimal sensitivity and sufficient feasibility data to form the basis for a large prospective study to guide radiotherapy decision-making. Greater collaboration between radiation oncology and commercial partners is required, as well as further investment into physician-scientist careers, given the complex nature of this technology.

## 5 Future horizons for ctDNA in radiation oncology

At present, there is clear evidence that ctDNA has a potential role in patient selection and risk-stratification for a range of pre- and post-RT scenarios in NSCLC. Integration of this molecular technology represents a new paradigm of opportunities whereby RT will be personalized. We beckon the interdisciplinary thoracic radiation oncology community to contemplate how ctDNA can be embedded in practice to advance the clinical management of NSCLC beyond current imaging-centric approaches [18]. Potential applications include selection of treatment paradigms, individualization of dose-fractionation, and follow-up personalization [19]. As geometry-based adaptive RT practice clinically matures, integration of ctDNA molecular response during treatment could also facilitate biology-based adaptive treatment [20]. Furthermore, the importance of normal tissue toxicity avoidance in radiation oncology [21] might be augmented by cell-free DNA methylation approaches, as recently suggested for cardiac, lung and liver toxicity following right-sided breast RT [22], and subclinical anthracycline cardiotoxicity [23]. As targeted drugs in combination with RT emerge as a new treatment paradigm [24], tracking dominant tumor subclones [25] could anticipate resistance and inform subsequent drug selection. Also would be the use of ctDNA to more precisely select patients for adjuvant immunotherapy following RT [26] or to measure radiation resistance signatures [27]. Finally, RT could be utilized to increase ctDNA assay sensitivity, as suggested by 50% of patients with undetectable levels having detectable levels post-fraction one [11,12]. Indeed, a ‘radiation-primed’ ctDNA measurement [10] in scenarios where a tumor biopsy is not possible could increase the range of detectable mutations, although the somatic mutational imprint of the radiation might need to be accounted for, along with risk for radiation toxicity [28].

In summary, there is a critical need for further inter-fraction ctDNA investigation in definitive NSCLC RT, given its potential to address the gaps in our current treatment paradigms. To achieve this, radiation oncologists and commercial technology companies should partner, and funding agencies should prioritize ctDNA RT-focused efforts. If successful, inter-fraction ctDNA could truly bring radiation oncology into the world of precision medicine.

## Funding statement

The authors did not receive any direct funding for this work. 10.13039/100007108GW holds a Visiting Fellow Scholarship from Fulbright Ireland and a Research Grant from the 10.13039/100005923International Association for the Study of Lung Cancer. AC 10.13039/100001368has R01 grant funding from the National Cancer Institute of the United States of America.

## Data availability statement

No original data were curated for this submission.

## Declaration of competing interest

The authors declare the following financial interests/personal relationships which may be considered as potential competing interests:

Gerard Walls reports a relationship with 10.13039/100004325AstraZeneca that includes: speaking and lecture fees. Bruna Pellini reports a relationship with 10.13039/100008021Bristol Myers Squibb that includes: consulting or advisory and speaking and lecture fees. Bruna Pellini reports a relationship with BioAscend that includes: consulting or advisory and speaking and lecture fees. Bruna Pellini reports a relationship with 10.13039/100004334Merck & Co Inc that includes: consulting or advisory and speaking and lecture fees. Bruna Pellini reports a relationship with MJH Life Sciences LLC that includes: consulting or advisory and speaking and lecture fees. Bruna Pellini reports a relationship with Play to Know AG that includes: consulting or advisory and speaking and lecture fees. Bruna Pellini reports a relationship with Grupo Pardini that includes: consulting or advisory and speaking and lecture fees. Bruna Pellini reports a relationship with GBOT that includes: consulting or advisory and speaking and lecture fees. Bruna Pellini reports a relationship with Doctaforum that includes: consulting or advisory and speaking and lecture fees. Bruna Pellini reports a relationship with Guidepoint that includes: consulting or advisory and speaking and lecture fees. Bruna Pellini reports a relationship with Guardant Health that includes: consulting or advisory and speaking and lecture fees. Bruna Pellini reports a relationship with Foundation Medicine that includes: consulting or advisory and speaking and lecture fees. Bruna Pellini reports a relationship with 10.13039/100010905Illumina that includes: consulting or advisory and speaking and lecture fees. Bruna Pellini reports a relationship with 10.13039/100009857Regeneron that includes: consulting or advisory and speaking and lecture fees. Bruna Pellini reports a relationship with 10.13039/100004325AstraZeneca that includes: consulting or advisory and speaking and lecture fees. Bruna Pellini reports a relationship with Merus that includes: consulting or advisory and speaking and lecture fees. Bruna Pellini reports a relationship with The Medical Educator Consortium that includes: consulting or advisory and speaking and lecture fees. Bruna Pellini reports a relationship with Medical Learning Institute that includes: consulting or advisory and speaking and lecture fees. Bruna Pellini reports a relationship with 10.13039/100008021Bristol Myers Squibb that includes: funding grants. Bruna Pellini reports a relationship with 10.13039/100008021Bristol Myers Squibb Foundation that includes: funding grants. Aadel Chaudhuri reports a relationship with 10.13039/100004337Roche that includes: consulting or advisory, funding grants, and speaking and lecture fees. Aadel Chaudhuri reports a relationship with Tempus that includes: consulting or advisory and funding grants. Aadel Chaudhuri reports a relationship with Geneoscopy that includes: consulting or advisory and equity or stocks. Aadel Chaudhuri reports a relationship with 10.13039/100010905Illumina that includes: consulting or advisory and funding grants. Aadel Chaudhuri reports a relationship with Invitae that includes: consulting or advisory. Aadel Chaudhuri reports a relationship with Myriad Genetics that includes: consulting or advisory. Aadel Chaudhuri reports a relationship with Daiichi Sankyo that includes: consulting or advisory. Aadel Chaudhuri reports a relationship with AstraZeneca that includes: consulting or advisory. Aadel Chaudhuri reports a relationship with AlphaSights that includes: consulting or advisory. Aadel Chaudhuri reports a relationship with DeciBio that includes: consulting or advisory. Aadel Chaudhuri reports a relationship with Guidepoint that includes: consulting or advisory. Aadel Chaudhuri reports a relationship with 10.13039/100004322Agilent that includes: speaking and lecture fees. Aadel Chaudhuri reports a relationship with Binaytara Foundation that includes: speaking and lecture fees. Aadel Chaudhuri reports a relationship with Droplet Biosciences that includes: equity or stocks. Aadel Chaudhuri reports a relationship with LiquidCell Dx that includes: board membership and equity or stocks. Aadel Chaudhuri reports a relationship with CytoTrace Biosciences that includes: board membership and equity or stocks. Aadel Chaudhuri has pending patents licensed to Droplet Biosciences. Aadel Chaudhuri has a pending patent licensed to Tempus Labs. Aadel Chaudhuri has pending patents licensed to LiquidCell Dx. Aadel Chaudhuri has a patent licensed to Biocognitive Labs. Given their roles in the journal, Bruna Pellini (Editorial Board Member) and Aadel Chaudhuri (Editorial Board Member) had no involvement in the peer review of this article and had no confidential access to information regarding its peer review.
